# Strong-correlated behavior of 4*f* electrons and 4*f*5*d* hybridization in PrO_2_

**DOI:** 10.1038/s41598-018-34336-4

**Published:** 2018-10-30

**Authors:** Lifang Zhang, Junling Meng, Fen Yao, Xiaojuan Liu, Jian Meng, Hongjie Zhang

**Affiliations:** 1 State Key Laboratory of Rare Earth Resources Utilization, Changchun Institute of Applied Chemistry, Chinese Academy of Sciences, 5625, Ren Min Street, Changchun, 130022 P. R. China; 2 University of Science and Technology of China, 96, JinZhai Road, Baohe District, Hefei, Anhui 230026 P. R. China; 3 University of Chinese Academy of Sciences, 19(A), Yuquan Road, Shijingshan District, Beijing, 100049 P. R. China

## Abstract

Bringing oxygen atoms from infinite, passing equilibrium until short enough distances, we aim to reveal the 4*f*5*d* electron bonding property and its relevance to the peculiar physical properties within PrO_2_ based on both accounting for electron Coulomb repulsion and spin-orbit coupling effects in combination with Wannier function methods. The microscopic mechanism of static Janh-Teller distortions and the physical insight into the dynamic Jahn-Teller effects are clarified. Peculiarly, the magnetic coupling is suggested to be via 4*f*-5*d*-O2*p*-5*d*-4*f* pathway in PrO_2_, and the coupling between spin and orbital ordering of 4*f* electrons is for the first time disclosed. The 5*d* orbitals, hybridized with 4*f* electrons, are found to play important roles in these processes.

## Introduction

The electron correlations in transition metal oxides 3*d* electrons constrain the number of electrons at a given lattice site, and induce a local entanglement of the charge, spin and orbital degrees of freedom. This gives rise to a variety of phenomena, for example, Jahn-Teller distortions, various charge, spin and orbital orderings, metal-insulator transitions, multiferroics and superconductivity^1,2^. Recently, Jahn-Teller distortions and orbital phenomena have also been observed in compounds containing localized 4*f* and 5*f* electrons. Among these, the actinide dioxides UO_2_ and NpO_2_ in fluorite-structure have been studied for many years^3,4^ and have been revealed to possess complex ordered phases at low temperatures involving coupled electric and magnetic multipoles as well as a lattice distortion (here in the case of UO_2_). For 4*f* electron-related models, lanthanide dioxide PrO_2_ has attracted more attention in recent years. In a separate neutron and x-ray diffraction measurements of crystallographic and magnetic structure in the temperature range of 2–300 K, Gardiner *et al*. revealed the existence of an internal static distortion of the fluorite structure below *T*_D_ = 120 K and a related distortion of the antiferromagnetic structure below *T*_N_ = 13.5 K^5^. This raises the question of what mechanism drives the structural distortion of PrO_2_. Two possible mechanisms have been put forward: one is that the electronic energy is reduced by a collective Jahn-Teller distortion at the expense of a small penalty in elastic energy, the other is that the distortion is a consequence of a quadrupolar ordering of the Pr 4*f* orbitals since there is evidence for a degree of Pr 4*f*-O 2*p* hybridization. Besides, taking PrO_2_ as a representative of the model system of dynamic Jahn-Teller effect, it has been probed by neutron inelastic scattering, providing strong evidence for dynamic Jahn-Teller effect in the Γ_8_ electronic ground state^6^. From these experimental evidences available so far it seems likely that both the static and dynamic Jahn-Teller effects are important in PrO_2_, but the microscopic physical insight has not been clarified. Another important issue that has never been noticed is whether the spin ordering is relevant to the orbital ordering within this strong correlated 4*f* system since spin-orbital orderings coupling could be widely observed in 3*d* electron transition metal oxides as mentioned above.

Using density functional theory (DFT) + *U* calculation, Fabien Tran *et al*. reproduced the structure of PrO_2_ with the Jahn-Teller distortion, coinciding with the experimental result rather well^7^. In ref.^8^, Jens Jensen reported the static and dynamic Jahn-Teller effects for PrO_2_ with a mean-field mode, which well described the properties of PrO_2_ in its paramagnetic phase. Also there are several density functional based studies describing the electronic structures based on self-interaction-corrected (SIC) local-spin-density approach^9^, the Jahn-Teller distortions in PrO_2_ via Perdew-Burke-Ernzerhof PBE + *U* calculations^10^, and dynamic Jahn-Teller effect by a simple model based on a vibronic Hamiltonian^6^, so far there is no microscopic insight into the versatile phenomena of PrO_2_ using a method that truly captures the underlying local physics. The maximally localized Wannier functions (MLWFs), with the linear combination of the delocalized molecular-orbital eigenfunctions corresponding closely to the “natural bond orbital”, could achieve this realization of the chemists’ imagination of localized bonds and lone pairs as basic units of molecular structure^11^. Thus, in our present study, combining DFT + *U* + SOC in with these Wannier-like orbitals^12,13^, we revisited the strong-correlation behavior of 4*f* electrons and the correlation with static Jahn-Teller distortion. Furthermore, according to the results of the 4*f*5*d* hybridization of PrO_2_ under a series of different distances (under different hydrostatic pressures), the dynamic Jahn-Teller scenery was visually figured out as supplying-conveying-reception model, which was one of progresses achieved in this work. Besides, the magnetic coupling between Pr ions was investigated and the coupling between spin-orbital orderings was also identified in this study. For the first time, a 4*f*-5*d*-O2*p*-5*d*-4*f* pathway for the electron exchange mechanism was explained the RKKY (Ruderman-Kittel-Kasuya-Yosida) interaction occurred in 4*f*-containing systems. The coupling between the spin- and orbital-orderings was indeed observed within different magnetic arrangements. Afterall, a comprehensive understanding of the strong correlated behavior of 4*f* electrons in PrO_2_ was given from a new point of view.

In brief, we attempt to address the following questions: (i) What is the mechanism of the static Jahn-Teller distortion? (ii) What is the physical scenery of dynamic Jahn-Teller effect and its origin? (iii) Is there spin-orbital ordering coupling within this 4*f* electron system as it exists in that of 3*d* electrons? (iv) The role of the 5*d* orbitals in these processes.

## Results

### Calculation Details

As we know, in the cubic fluorite structure of PrO_2_, the atomic coordination, including those of Pr and O atoms, are fixed leaving the lattice parameter (*a*) as the only degree of freedom. Thus, in this calculation, we take a series of different values of lattice parameter (*a*) to simulate the different distance between Pr and O, also to model the situation under different hydrostatic pressures. Through the detailed analysis of the electronic structure evolution along this variable value, the strong correlated behavior of 4f electrons and the 4f5d hybridization within PrO_2_ are expected to be figured out. We used the self-consistent DFT in generalized gradient approximation (GGA)^14^ with PBE_sol functionals (revised PBE for the solid)^15^ to obtain the structural parameters within the implementation of Wien2k package^16,17^. For strongly correlated 4*f* electrons, we simultaneously took the Coulomb repulsion (*U*)^18,19^ and spin-orbit coupling (SOC) into accounts in the electronic structure calculations. The double-valley-potential model observed in pervious X-ray absorption studies^20,21^ is then perfectly simulated. Different methods were also compared, such as hybrid functional method, and the relevant results were organized in Supplementary Notes 1–3. Furthermore, we also carried out calculations with the Wannier90 code^12^ to transform the localized and extended orbital into maximally localized Wannier functions (MLWFs) with the disentanglement procedure. Interestingly, with the combination of DFT and MLWF results, we took an insight into intrinsic characters of the physical phenomena which originated from the electronic correlations of 4*f* electrons and the hybridization between Pr-4*f*/5*d* and O-2*p* states. Since the magnetoelastic interaction is considered to participate at lower temperature at least where the magnetic coupling emerges, within our present comparatively large variable range, the magnetic coupling is omitted. Thus, we carried out the calculations in the ferromagnetic state excepting that all the magnetic configurations were taken into account in the last section within which the spin-orbit ordering coupling was investigated.

### Electronic structure at equilibrium geometry

**Figure 1:**
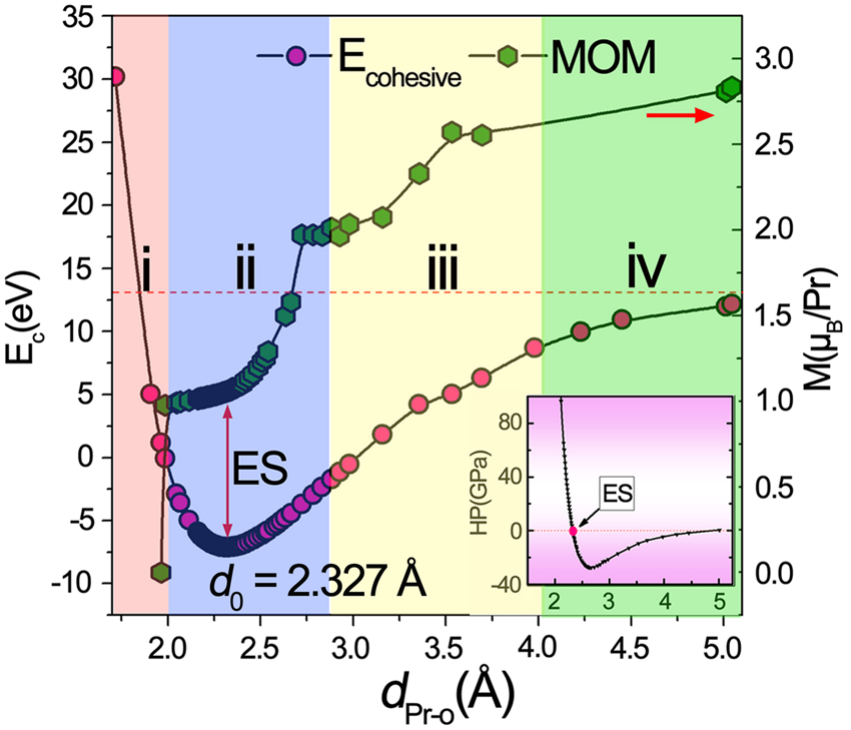
The cohesive energy curve varies with interatomic distance *d*_Pr-O_. The evolution of the cohesive energy *E*_c_ (left column) and the magnetic moment of Pr (right column) with the dependence of interatomic distance *d*_Pr-O_, and the insert plot represents the estimate of hydrostatic pressure accordingly. The whole variation range is divided into four parts, labeled by i, ii, iii and iv.

**Figure 2:**
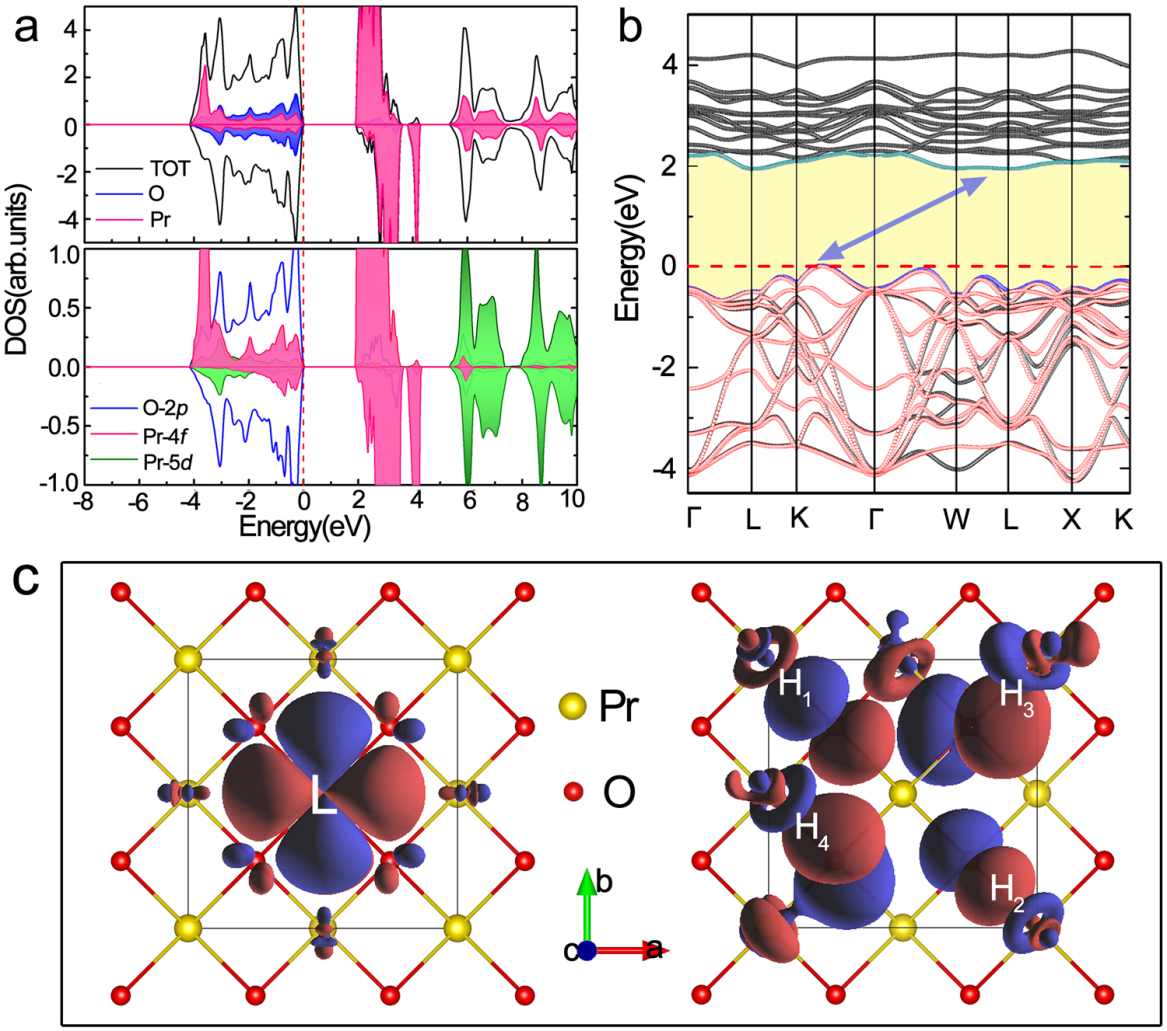
The electronic structure of the equilibrium PrO_2_. (**a**) The total and the partial density of states (DOS) in a reduced cell. (**b**) DFT + *U* + SOC computed band structure (black lines) of PrO_2_ together with the tight-binding bands obtained from MLWFs (red lines) at the valence band range. (**c**) The valence-bands Pr-4*f* Wannier orbitals for PrO_2_ visualized as an isosurface of the charge density |*w*(r)|^2^ (red = positive, blue = negative). The left for the localized orbital 4*f*z(x^2^ − y^2^), magnetic quantum number m = 2, and the right for the hybridized section of other Pr-4*f* orbitals bonging with O-2*p*, H_1_ = *f*(xz^2^ + yz^2^) (m = ±1), H_2_ = *f*[x(x^2^ − 3y^2^) + y(3x^2^ − y^2^)] (m = ±3), H_3_ = *f*z^3^ (m = 0), H_4_ = *f*xyz (m = −2). H_1_ and H_2_ are the formation of sigma σ bond, H_3_ and H_4_ are pi π bond. To interpret clearly, we use the suitable amplitudes of the contour surface for each of orbital respectively.

The typical cohesive energy curve is obtained by bringing O atom from infinite distance, passing equilibrium geometry until close enough to Pr under fluorite structure as shown in Fig. 1, from which the equilibrium distance is identified at *d*_0_ = 2.327 Å, in good agreement with the experimental value of 2.332 Å^5^. Based on this structure, we calculated the atomic- and orbital-projected electronic density of states (DOS) (Fig. 2a) and its corresponding band structure (Fig. 2b with black line) using GGA + *U* + SOC method. Below Fermi level, the Pr-4*f* (pink shadow) states include a sharp peak at −3.75 eV and a broad peak centered around −1.5 eV extending from −3.5 to 0 eV. This double-valley-potential shape reflects the coexistence of localized and hybridized states. The evidence of a degree of Pr 4*f*/5*d*–O 2*p* hybridization in PrO_2_ implies the entanglement of electronic correlation. Probed by neutron inelastic scattering, a broad continuum in magnetic excitation spectrum was discovered by Boothroyd and co-workers^6^, with which our electronic structure is even semi-quantitatively agreed. Since a cooperative Jahn-Teller distortion was observed in single crystal PrO_2_ at *T*_D_ = 120 ± 2 K^5^, as an insulator with a simple structure and localized 4*f* electrons, PrO_2_ seems an ideal model system for studies of Jahn-Teller effect containing 4*f* electrons. It is well known that Jahn-Teller fluctuation contains the orbitally degenerate 4*f* ground state and dynamic distortion of lattice. Therefore, understanding the electron correlations at equilibrium position is a fundamental task to seize the static Jahn-Teller mechanism which will be presented in the following.

### Static Jahn-Teller distortion

**Figure 3:**
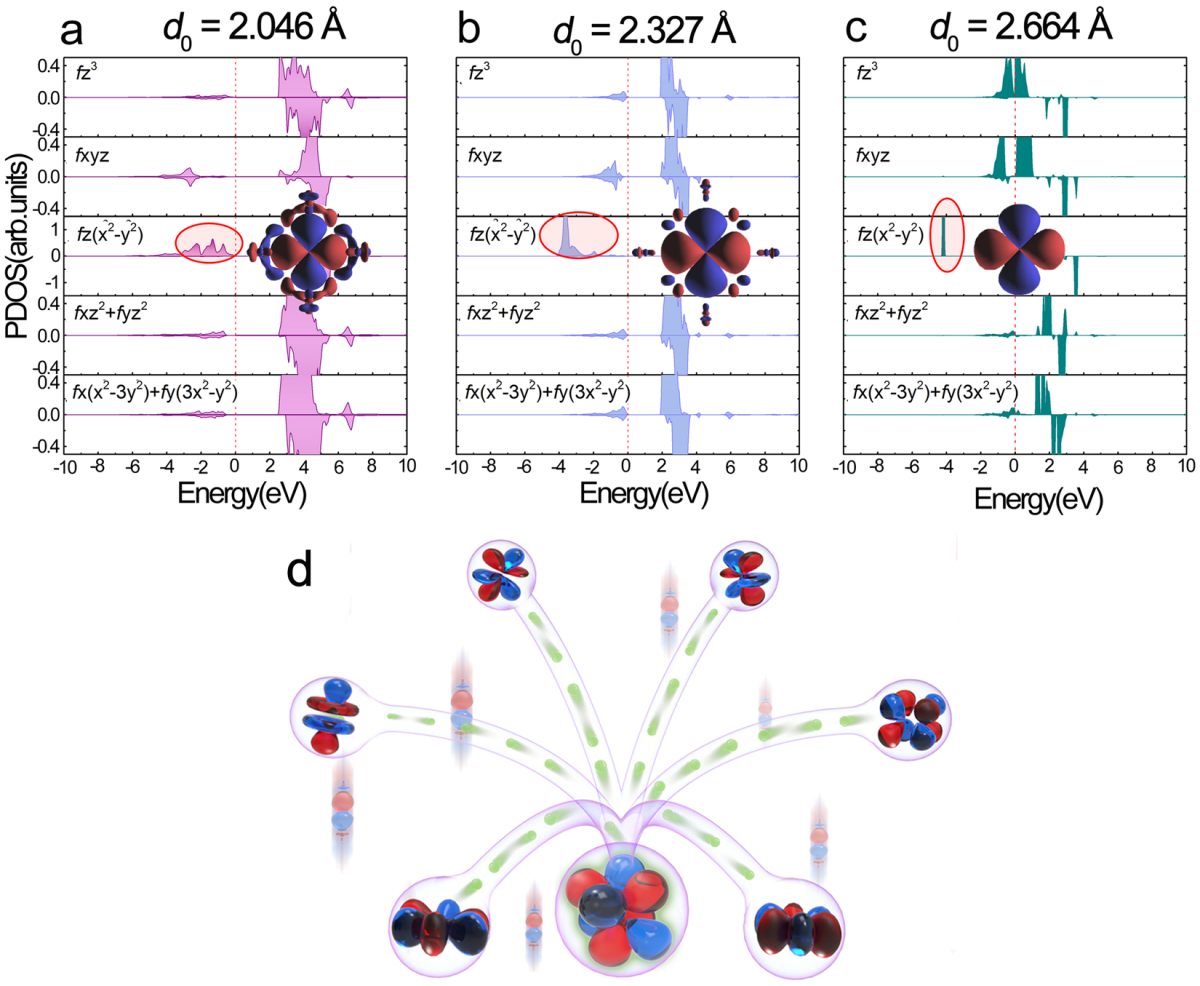
Transformation from localization to delocalization. (**a**–**c**) Delocalization-localization transformation exists in area ii: the PDOS of the 4*f* orbitals for PrO_2_ with *d*_Pr-O_ = 2.046, 2.327 and 2.664 Å respectively. We show the valence-band MLWFs for *f*z(x^2^ − y^2^) at the same isosurface values ± 0.0002 Å^−3/2^. (**d**) Schematic diagram showing the strongly correlated Pr-4*f*/5*d* electrons that can represent the mechanism of the transformation of 4*f*z(x^2^ − y^2^) from delocalization to localization.

Since the Pr-4*f* orbitals play a vital role in Jahn-Teller distortion, we examine the electronic structures at equilibrium fluorite structure of PrO_2_. Figure 3b presents the orbital-projected partial density of states (PDOS) of Pr-4*f* orbitals. Under the Fermi level at lower energy of ~3.75 eV, *f*z(x^2^ − y^2^) orbital (magnetic quantum number m = 2) is fully occupied as a localized state, which reflects a doublet for the crystal field ground state, being consistent with the lowering of the local symmetry of Pr. However, for the other six 4*f* orbitals, on one hand, there are two pairs of degenerate orbitals: (*f*xz^2^ + *f*yz^2^) and [*f*x(x^2^ − 3y^2^) + *f*y(3x^2^ − y^2^)], on the other hand, we notice that the left electrons are seemingly equally distributed on these orbitals except that *f*xyz orbital is slightly preferentially occupied. Recalling that Mn^3+^ with *d*^4^ configurations at a prefect octahedral crystal field, the degenerate *d*x^2^ − y^2^ and *d*z^2^ orbitals are inclined to split under a distorted octahedral field^22^, it is similarly concluded that these degenerate 4*f* orbitals and the 4*f* electrons occupied in degeneration demonstrate the instability of electronic structure in this configuration. It is accepted that the undistorted PrO_2_ in this state is unstable in some degree, which has been observed in experimental previously^5^. Thus, this electronic instability is supposed to be related to the structural distortion at the expense of small penalty in elastic energy^5,6^.

It has been well known that Wannier functions can serve as a useful tool to allow the chemical bonds in the periodic systems to be directly visually analyzed, especially in the solution of the problem of electron correlation systems^23^. With the aim to clearly capture the local physical insight of the static Jahn-Teller distortion in PrO_2_, we intent to construct the complex Wannier orbitals for Pr-4*f* states, by which the obtained tight-binging bands (highlighted with red lines) are consistent with the corresponding band structure calculated by self-consistent DFT + *U* + SOC as shown in Fig. 2b. In more details, Fig. 2c in the left shows that the localized 4*f* orbital with m = 2 has eight lobes with their angular moments direction maximally deviating away from oxygen atoms, being agreed with the observation by Tran *et al*.^7^. This occupied state donates the overall stability of 4*f*^1^ configuration for PrO_2_ in the cubic fluorite octahedral crystal field. Nevertheless, the case of the covalent hybridized part around ~−1.5 eV is somehow complex, whose Wannier orbitals analysis is shown in the right of Fig. 1c. Although O-2*p* states are largely predominant in valence bands, a certain proportion of Pr-4*f* and 5*d* electron states (Fig. S5c in Supplementary) are simultaneously visible. Particularly, we have noticed two types of spatial orientations between Pr-4*f* and the O-2*p* orbitals (see the right of Fig. 2c)^24^: one is a head-on overlapping sigma σ bonding enclosed by H_1_ and H_2_, and the other is a sideways overlapping π bonding labeled by H_3_ and H_4_. Furthermore, in more quantitative analysis it is found that the proportion of π bond is larger than that of σ bond by computing the occupation number for each 4*f* electrons. Since π bonds are obviously weaker than σ bonds, it indicates a tendency to a distortion that further lowers the total energy. We conclude that this is the microscopic mechanism of the Jahn-Teller distortion which should lead to more head-on σ bonding. As to what extent this distortion will be, there exists a balanced factor that needs to be taken into account. On one hand, this distortion will lower the total energy by enhancing the head-on σ bonding, simultaneously, the electrostatic interactions for the localized stable states and the existing head-on σ bonding (H1 and H2) will disadvantageously increase. Therefore, we conclude that the extent of static Jahn-Teller distortion in PrO_2_ is attributed to the synergistic effect of the localization and hybridization originating from the strong correlated 4*f* electrons.

### Dynamic Jahn-Teller effect (localization-delocalization transformation)

The cooperative Jahn-Teller effect is a phase transition which is driven by the interaction between the electronic states and crystal lattice^25^. To our acknowledgement, the static Jahn-Teller distortion in PrO_2_ via the internal O displacement derives from the change of 4*f* electron crystal-field Hamiltonian, while the dynamic Jahn-Teller effect which mixes the electronic and phonon coordinates involves a magnetoelastic coupling of the phonons and the magnetic excitation^8^. In order to overview the whole picture of the dynamic Jahn-Teller effect, we detect the variation of the detailed electron structures with the external perturbation by the application of hydrostatic pressures. The prototypical cohesive energy curve, representing the interaction^26^ between two atoms varies with interatomic distance *d*_Pr-O_, is obtained as shown in Fig. 1. Basically, for *d*_Pr-O_ > *d*_0_, the total energy increases gradually, approaching a constant as *d*_Pr-O_ → ∞, while for *d*_Pr-O_ < *d*_0_, the energy increases exponentially, approaching ∞ at a sufficiently small interatomic distance. According to the magnetic moment of Pr, we divided the whole process into four subsections labeled by i, ii, iii and iv respectively, as displayed in Fig. S4 in the Supplementary.

We pay more attention to the electronic structure evolution within subsection ii where *d*_Pr-O_ ranges from 2.0 Å to 2.7 Å around the equilibrium distance due to the fact that it is more experimentally accessible compared to the other three subsections. Previous neutron spectra demonstrated a variation of the broad band of vibronic scattering with the dependence of temperature, which had been attributed to a dynamic Jahn-Teller interaction^27^. Analogously, in Fig. 3a–c combined with Fig. S5 in Supplementary, we presented the 4*f*- and 5*d*-projected PDOS calculated at *d*_Pr-O_ = 2.046 Å, 2.327 Å and 2.664 Å within which *f*z(x^2^ − y^2^) orbital obtained via Wannier constructions were inserted. In details, at the equilibrium distance of 2.327 Å, the localized *f*z(x^2^ − y^2^) orbital at around ~−3.75 eV display little connection with the delocalized part at around ~−1.5 eV; while as the *d*_Pr-O_ become smaller, here refer to 2.046 Å, accompanying the rise of orbital energy, on one hand a portion of localized electrons within the localized *f*z(x^2^ − y^2^) orbital become delocalized, on the other hand the localized orbital looks seemingly delocalized, completely connected between the localized and delocalized components; On the contrast, when *d*_Pr-O_ is as large as to be 2.664 Å, this localized *f*z(x^2^ − y^2^) orbital is fully occupied at a lower energy level with no connection with the delocalized part any more, during which accompanies the backflow of the delocalized electrons into the localized *f*z(x^2^ − y^2^) orbital. Apparently, within our research, taking interatomic distance as an external perturbation, the electrons localization-delocalization transformation is represented, and the strong coupling between 4*f*^1^ electronic states and local dynamic lattice distortions have been figured out. These results are consistent with the literature^25^, and it is concluded that this coupling causes the dynamic Jahn-Teller effect in the Γ_8_ ground state and simultaneously provides us the full physical scenery for the dynamic Jahn-Teller effect.

According to the detailed detection for each electronic structures of PrO_2_ in subsection ii, quantitative analysis of the occupancies of orbitals and their energy center levels relative to Fermi level have been summarized in Figs S6 and S7 in the Supplementary. From Fig. S6, we can see that the occupancy number of the localized *f*z(x^2^ − y^2^) gradually increases while the occupancy number of the delocalized section decreases along with the lengthening of *d*_Pr-O_ (see Fig. S6a). For the case of 5*d* orbitals, the change of occupation number (see Fig. S6b) is comparatively weak to that of 4*f*. From Fig. S6d, it is noticed that the energy center level of the localized 4*f* orbital decreases and that of the delocalized part moves towards Fermi level. From Fig. S6e, we can see that the energy center levels for 5*d* orbitals shift gradually towards Fermi level with the increase of *d*_Pr-O_. The detailed analysis of the change tendency for angular-projected PDOS of 4*f* orbitals is shown in Fig. S7, it can be seen that the change within *f*z(x^2^ − y^2^) orbitals represents the main tendency for both occupation number and the energy level relative to the Fermi level. There seems exist a discontinuity of the spin-down channel in Fig. S7c, here we need to make this be clarified. In fact, the changing tendency is continuous if we add all the parts including A_n_, B_n_, and C_n_ as shown Fig. S8 in the Supplementary. However, we have counted only the highest peak under different situation, this leads to the superficial discontinuity. All these results indicate that there exists a quasi-continuous delocalization-localization transformation for this strong-correlated 4*f* system within this subsection ii: at the small *d*_Pr-O_, the hybridization between O and Pr ions enhances, the localized 4*f* state, supplies electrons for the delocalized ones through the 5*d* channel; while as the increase of *d*_Pr-O_, some of the delocalized covalent 4*f* electrons flow back to this ‘reservoir’ via the 5*d* connection until the localized one reaches saturation. During this transformation, the localized 4*f* state acts as electron reservoir for the delocalized 4*f* electrons, and the 5*d* bands play the important role of the pipeline (see Fig. 3d). As the data indicates, the energy center levels of both 4*f* and 5*d* orbitals continuously alter with *d*_Pr-O_ and their relative positions dynamically change. At the same time, from the analysis above, we can see that 5*d* orbitals act as pipeline during this transformation, thus the position of the 5*d* orbitals must be positioned among the localized and delocalized energy range. Accordingly, out of this scope, the localization-delocalization transformation would not occur due to the absence of the 5*d* pipeline. Indeed, the electronic analysis above has revealed that the connection by 5*d* states is broken off in the other three subsections: when *d*_Pr-O_ is too small, 5*d* bands move below the 4*f* energy level; when *d*_Pr-O_ is large enough, the 5*d* electrons move above 4*f* states.

### Magnetic interaction mechanism and the coupling between spin-orbital ordering

**Figure 4:**
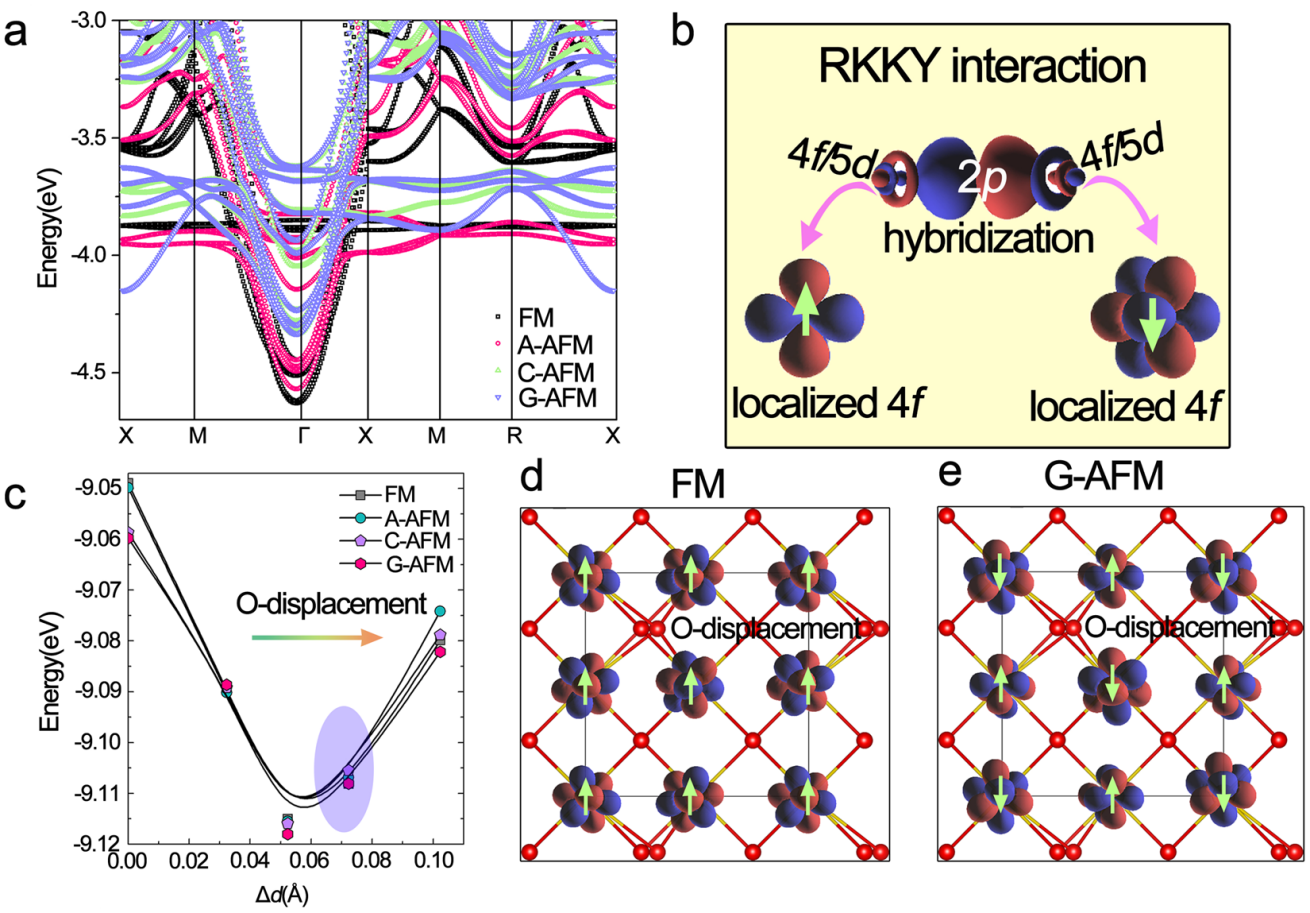
Magnetic orderings dependence of the strong-correlated behaviors in 4*f*/5*d* electrons. (**a**) The tight-binding bands for PrO_2_ with different magnetic structures, which in the energy region [−5.0 eV, −3.0 eV] indicates the contribution of the 4*f* localization, 2*p*-5*d*-4*f* hybridization from bottom to top. (**b**) Schematic of rare-earth magnetic superexchange-like interaction: the spin-orbit ordering coupling between 4*f* electrons is mediated by the Pr-4*f*-5*d*-O-2*p* delocalization. (**c**) PBE_sol + *U* + SOC (*U*_eff_ = 6 eV) cohesive energy vs distortion Δ*d* form the perfect cubic fluorite structure and the Jahn-Teller distorted structures, the shadow region represents the range of experimental values. (**d**,**e**) The plots of localized 4*f* Wannier orbitals for the distorted PrO_2_ (Δ*d* = 0.0723 Å) with FM and G-AFM magnetic configurations, the amplitudes of the contour surface are ± 0.01 Å^−3/2^.

The magnetic moments between Pr have been confirmed to be antiferromagnetically coupled at *T*_N_ = 13.5 K with a type-I magnetic structure as found in UO_2_^28^. In order to investigate the magnetic interaction mechanism in PrO_2_, four different magnetic structures: ferromagnetism (FM), A-type-antiferromagnetism (A-AFM), C-type-AFM and G-type-AFM were designed, similar to the case in LaMnO_3_ system. Probed the magnetic stability via the comparison of their total energies, it is found that the G-AFM structure is the most energetically favorable, while FM is the most unfavorable, indicating that Pr magnetic moments are prone to antiferromagnetically coupled in all the three directions. Figure 4a shows the tight-binding bands for PrO_2_ with different magnetic orderings. In the energy region [−5 eV, −3eV], it seems that the localized 4*f* electrons play a predominant role in the magnetic stability, which the AFM structures lower their energy by splitting the 4*f* orbitals into different energy levels, while in FM structure, the degree of 4*f* degeneration rises with the increasing of total energy. It has been well known that the magnetic interaction between rare earth metals is RKKY interaction, which is coupled via an indirect exchange interaction involving the conduction electrons^29,30^. Thus, an issue emerges that what is the mediate interaction of the RKKY interaction in this dioxides PrO_2_. Figure 4b shows schematic of the magnetic interactions obtained by Wannier function. Therein, we can see that Pr atoms interact with one another via the nonmagnetic oxygen atoms between them similarly between the transition metals in the transition metal oxides. However, unlike itinerant 3*d* systems such as MnO^31,32^, 4*f* electrons couple via indirect exchange interactions mediated by their 4*f*/5*d* hybridization, *i.e*. with a 4*f*-5*d*-O2*p*-5*d*-4*f* pathway, since the overlap between the single localized Pr-4*f* electron and the electrons of the surrounding oxygen is weak. Since then, it is believed that the crystal parameters, such as the Pr-O bond length and Pr-O-Pr bond angles, are important factors for mediating the superexchange-like interaction between the 4*f* electrons within this RKKY interaction.

On the other hand, it has been well known that within transition metal oxides, magnetic ordering is strongly coupled with the ordering of the occupied/unoccupied electronic states (orbital ordering) controlled by the Jahn-Teller distortions^33,34^. For 4*f* electrons, which are considered to be shielded in the chemically inert core, we are intent to inquire whether the coupling between spin ordering and orbital ordering still occurs. First, based on the analysis of Wannier function at the undistorted fluorite structure, we obtained the orbital ordering at different spin arrangements including FM, A-AFM, C-AFM and G-AFM as shown in Fig. S9 in Supplementary. It is observed that different magnetic couplings only lead to different angular momentum linear combination while the preferential occupation for a certain orbital does not occur. In particular, since the orbital ordering is controlled by Jahn-Teller distortion, we then artificially move the oxygen atoms according to the Wannier function analyzed results above, as shown in Fig. 4c, the total energy reaches the minimum with the displacement ~0.059 Å, which is only 0.006 Å smaller than the lower bound of the range 0.065–0.078 Å of the experimental values^5^ and agree well with other calculated results^22^. Then, we purposely calculated the orbital orderings by Wannier function based on the distorted structures at FM and G-AFM magnetic configurations. Again, it is found that different magnetic couplings only lead to different angular momentum linear combination while the preferential occupation for a certain orbital does not occur even at these distorted structures as shown in Fig. 4d,e with Fig. S10 in the Supplementary presenting a more detailed presentation. It is therefore concluded that the spin-orbit ordering coupling within 4*f* electrons acts remarkably different from the case of 3*d* electrons, the magnetic coupling only leads to the different angular momentum linear combination without an obvious preferential occupation at certain orbitals. We attribute this to the fact that 4*f* electrons are shielded in the chemically inert core which is insensitive to the external simulations.

## Discussion

Thus, in this article we present the strong correlated behavior of 4*f*^1+^ electrons (plus as the partially filled electron) in PrO_2_ under octahedral crystal field and its correlation to the static and dynamic Jahn-Teller distortions, magnetic couplings and spin-orbital ordering coupling. It is found that similar to strong-correlated 3*d* electrons, the entanglement of charge, spin and orbital orderings, Jahn-Teller distortions have been similarly discovered in 4*f* electron systems. Based on this research, we expect that systematic investigations on the strong correlated behavior of 4*f* electrons should be carried out considering the effect of both crystal field and the different electronic configurations (from 4*f*^1^ ~ 4*f*^13^, that is from Pr^3+^ ~ Yb^3+^) using such as the group theory. It is believed that these complete data should be absolutely indispensable to further design versatile advanced rare earth functional materials.

The strong correlated behavior of 4*f* electrons in PrO_2_ has been investigated based on the GGA + *U* + SOC and Wannier function method herein. Our results show that the static Jahn-Teller distortion is induced by the degeneracy of 4*f* orbital and is contributed by the synergistic effect of the localization and hybridization. The dynamic Jahn-Teller simulation indicates a localization-delocalization transformation for *f*z(x^2^ − y^2^) electron, and reveals the electron supplying-conveying-reception relationship among the localized 4*f* electron, small occupied 5*d* electrons and delocalized 4*f* electrons. Moreover, introducing the magnetic orientations into the equilibrium PrO_2_, a 4*f*-5*d*-O2*p*-5*d*-4*f* pathway for the electron exchange mechanism explained the RKKY interaction occurred in 4*f*-containing systems, which seemingly likes the 3*d* superexchange interaction. Simultaneously, the coupling between the spin- and orbital-orderings is indeed observed within different magnetic arrangements while it acts remarkably different from the case of 3*d* electrons, the magnetic coupling only lead to the different angular momentum linear combination without obvious preferential occupation at certain orbitals. Thus, our article gives us a comprehensive understanding with the behaviors of strongly correlated 4*f* electrons in PrO_2_. However, the further work is required to disentangle the tanglesome factors for 4*f*-containing systems both on advanced theoretical detections and decisive experimental evidence.

## Electronic supplementary material

Supplementary information
