# Supplementary material for: Strong-correlated behavior of 4f electrons and 4f5d hybridization in PrO2
Source: Sci Rep. 2018 Oct 30;8:15995. doi: 10.1038/s41598-018-34336-4 (PMC6207729; doi:10.1038/s41598-018-34336-4)
Supplement: Supplementary file 1 — Supplementary information [file 41598_2018_34336_MOESM1_ESM.pdf]

Supplementary information:

Strong-correlated behavior of 4*f* electrons and 4*f*5*d* hybridization in PrO_2

Lifang Zhang,[1,2] Junling Meng,[1] Fen Yao,[1,3]Xiaojuan Liu,[1,2,*] Jian Meng,[1,2] Hongjie Zhang,[1,2]

[1] State Key Laboratory of Rare Earth Resources Utilization, Changchun Institute of Applied Chemistry, Chinese Academy of Sciences, 5625, Ren Min Street, Changchun, 130022, P.R.China.

[2] University of Science and Technology of China, 96, JinZhai Road, Baohe District, Hefei, Anhui, 230026, P.R.China.

[3] University of Chinese Academy of Sciences, 19(A), Yuquan Road, Shijingshan District, Beijing, 100049, P.R.China.

[*] Correspondence and requests for materials should be addressed to X. L. (email: lxjuan@ciac.ac.cn)

Supplementary Note 1: Calculation details

So far, the first-principles calculations have proved central in predicting new advance materials and/or explaining their behavior mechanism, many times leading to subsequent experimental observation[1,2]. However, it is hard to describe the strongly correlated systems containing transition metals with *d* electrons, lanthanide and actinide elements with *f* electrons based on standard density function theory (DFT) method. Hence, many groups are tending to promote the algorithm to remedy the shortcomings in theoretical predictions, such as the self-interaction correction local spin density (SIC-LSD) approach[3], DFT + *U* (onsite Coulomb interaction)[4,5], the hybrid DFT functional[6,7], and DFT + DMFT (dynamic mean-field theory)[8,9]. Based on these newly developed methods, numerous meaningful theoretical research on *f* orbitals concerned materials have been performed. For instance, based on the SIC-LSD approach, A. Svane's group studied the valency property of REs clearly[10], and in their latter work, they perfectly described the electronic structures of $PuO_{2\pm x}$[11] and the magnetism of heavy RE elements[12]. F. Tran and co-workers simulated the Jahn-Teller effect in PrO_2 with DFT + *U* with spin-orbit coupling (SOC) and their results were in line with experimental observations.[13] Lately, using the hybrid DFT functional, T. Duchon *etc.*[7] showed that the *f* contribution in CeO_2 was of covalent nature with experimental evidence. All in all, we keep our minds on that all calculating methods must serve for the first-principles researches and all researches must be faithful to the scientific facts. Our calculations were carried out by the WIEN2k code which was based on spin-polarized DFT with the full-potential (linearized) augmented plane-wave and local orbitals [FP – (L) APW + lo] method.[14-16] During structural optimization, to obtain the equilibrium PrO_2 structure, which is approximate to experimental result, we carried out kinds of the exchange correlation potential a *k*-point grid of size 7 × 7 × 7, and all structural data are estimated by the Murnaghan-Birch equation of states fitting,[17] as the example shown in Fig. S1. Compared

the fitted lattice constant (a) and bulk modulus (B) with experimental data, gathered in Table. SI, we found that the result computed by the generalized gradient approximation (GGA)[18,19], formulated by Perdew-Burke-Ernzerhof for solid (PBE_sol) functional[20], was more reasonable as the equilibrium structure. $R_{MT}^{min} K_{max} = 9.0$ (the smallest of the muffin-tin radii R_{MT} and the plane-wave cutoff parameter K_{max}) was used as basis set since the test calculations. We implemented Vienna *ab initio* Simulation Package (VASP)[21,22] to systematically estimate the total energy and so-called hydrostatic pressures, depended on the functional interatomic distance $d_{Pr\text{-}O}$. The cohesive energy (E_c) was calculated with the formula:[23]

$$E_c = (E_{TOT}^{d_{Pr-O}} - 4E_{Pr} - 8E_O)/4$$

where $E_{TOT}^{d_{Pr-O}}$ is the total energy of PrO_2 with difference $d_{Pr\text{-}O}$, E_{Pr} and E_O refer to atomic potential energy of Pr and O, respectively.

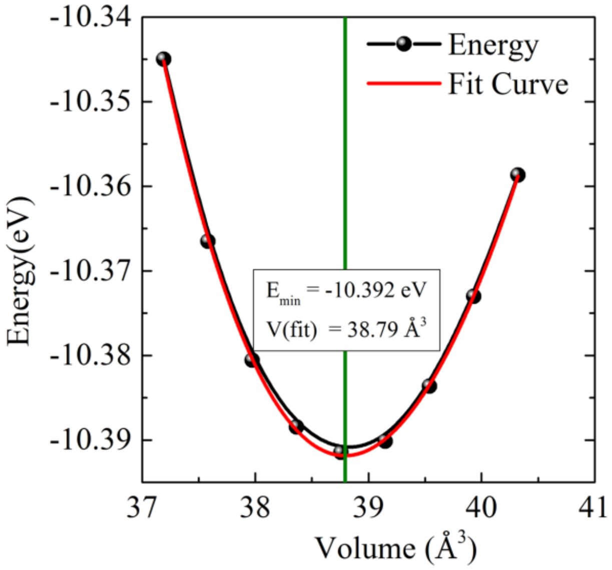

Supplementary Figure 1: **The fitting curve with the Birch–Murnaghan equation of state for equilibrium PrO_2 based on PBE_sol functional.** The equilibrium structures within different pseudo-potentials were obtained by this equation of state.

Supplementary Table 1: Equilibrium lattice constant volume and bulk modulus obtained by the different DFT methods without *U* and experiments.

Methods	*a* (Å)	Volume (Å^3)	*B* (GPa)
LDA	5.3312	37.8698	203.7574
PBE	5.4558	40.5872	163.8746
PBE_sol	5.3741	38.7923	199.7253
B3LYP	5.4573	40.6321	146.8626
Exp[a]	5.386	39.4	187

[a]Ref. 24

Supplementary Note 2: Test for optimization method

As we know, the equilibrium properties, such as bulk modulus (*B*), phonon frequencies, magnetism and ferroelectricity, are sensitive to the lattice constant *a*. PrO_2 possess a cubic-fluorite crystallographic structure. In order to be agreement with the experimental lattice constant of PrO_2 (a = 5.386 Å)[24], we have preformed DFT calculations with many exchange-correlation functionals for the structural optimization. Here *U* is not taken into consideration. Each of structure has reached the minimized ground state energy with the fitting of Birch–Murnaghan equation of state (BM-EOS), as the example displayed in Fig. S1. Their results are summarized in Table. SI. The volume is underestimated and bulk modulus is correspondingly overestimated obtained by LDA functional owing to its description to over bind atom. On the other hand, the equilibrium properties computed by Perdew-Burke-Ernzerhof (PBE) and B3LYP formulas are overcorrected as anticipated. It is proved that the revised PBE GGA, *i.e.* PBE for solid (PBE_sol) that improves equilibrium properties of densely packed solids and their surfaces. So with the PBE_sol functional, our calculated

equilibrium structure ($a_{PBE\text{-}sol}$ = 5.3741 Å) is well agreement with the experimental observations.

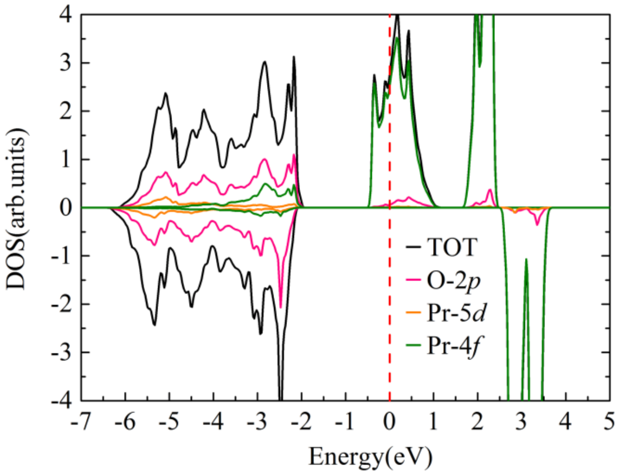

Supplementary Figure 2: **The electronic density of state (DOS) for PrO_2 based on the hybrid functional calculation**. The setting of $\boldsymbol{\alpha}$ = 0.25, $\boldsymbol{\lambda}$ = 0.165 $Bohr^{-1}$ was similar to HSE06 hybrid functionals.

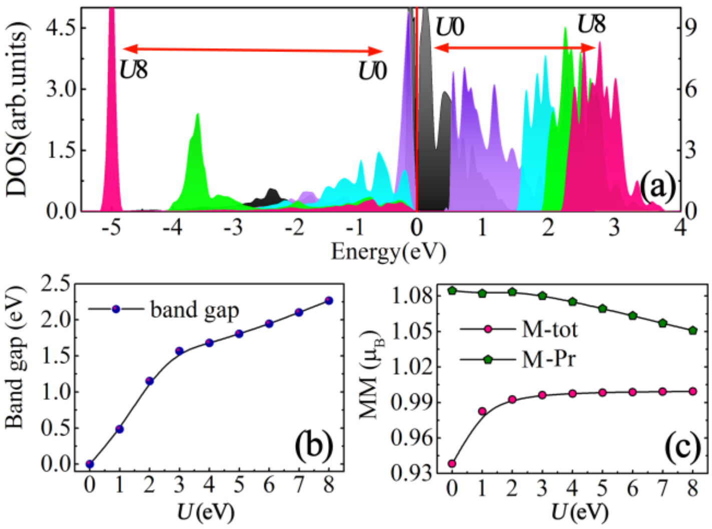

Supplementary Figure 3: Calculated properties of equilibrium PrO_2 based DFT with different *U*. (**a**) The partial density of states (PDOS) for the occupied (below Fermi energy) and empty (above Fermi energy) 4*f* orbitals. (**b**) Band gap and (**c**) Magnetic moment with dependence of *U*.

Supplementary Note 3: An accurate method to describe the electronic structure

In order to consistent the reported nature of PrO_2 well, especially the strongly correlated (localized) 4*f* electrons, we used hybrid functionals[25] and GGA + *U* methods to implement the self-consistent-field (SCF) calculations. All the convergence of the SCF calculations is attained with a charge convergence tolerance less than 10^{-4} Ry and 1000 *k*-points in the whole Brillouin zone. Although tested the different exact non-local exchange ($\boldsymbol{\alpha}$ = 0.1, 0.2, 0.25, 0.3, 1) and screening parameters ($\boldsymbol{\lambda}$ = 0, 0.053, 1.06, 1.65 $Bohr^{-1}$) in the hybrid functionals calculations[25], we found that the metallic behavior in equilibrium PrO_2 could not be avoid, (see the Fig. S2) which were conflictive to the insulate nature of PrO_2. However, the problem was solved out by introducing a strong intra-atomic interaction in a screen Hartree-Fock like manner, *i.e.* *U* into GGA. It is accepted that the U_{eff} ($U_{eff} = U - J$, setting $J = 0$) is treated as an empirical fitting parameter to make the simulate values approach to experiments. So, to find out a suitable U_{eff} in Pr-4*f*, we tested the value of U_{Pr} from 0 to 8 eV. Summarized each results (their density of states, band gaps and magnetic moments as shown in Fig. S3) with the previous studies, we set U_{Pr} = 6 eV in all of electronic structure calculations, not only for equilibrium state of PrO_2 with different magnetic orderings but also for $d_{\mathrm{Pr\text{-}O}}$ functional systems. The SOC was taken into consideration in the GGA + *U* calculations based on the second variational approach. In order to describe the 4*f* electrons clearly, we used the Wannier90 code[26] for the generation of maximally localized Wannier functions[27,28] in a direct space with visualization.

Derived from experimental data, Van der Kolk and Dorenbos proposed a model to predict the insulating, semiconducting or metallic behaviors, nature, band gap and chemical stability of lanthanide materials.[29] According to this model the undistorted PrO_2 was predicted to be an insulator with a band gap of ~ 2.0 eV, and furthermore the measured onset of the optical conductivity of $PrO_{2-\delta}$ was ~ 2.0 eV as well. As is well-known that it is hard for

the standard DFT describe the strongly correlated systems correctly such as compounds containing transition metals 3*d* or lanthanide elements 4*f*. The DFT calculations failed to open a gap and indicated an insulating phase for PrO_2 as expected. Surprisingly, the hybrid functional calculations with the different exact non-local exchange ($\boldsymbol{\alpha}$ = 0.1, 0.2, 0.25, 0.3, 1) and screening parameters ($\boldsymbol{\lambda}$ = 0, 0.053, 1.06, 1.65 $Bohr^{-1}$) describe a semi-metallic behavior which is inconsistent with the natural insulating property of PrO_2 as well. The density of states (DOS) obtained with hybrid functionals calculations (one example see in Fig. S2) indicates that the Pr-4*f* states are situated at Fermi surface and there is no atomic-like 4*f* electron localized below the Fermi level. However, the portion of which hybridization between O-2*p* and Pr-4*f*/5*d* is similar to the previous calculations. The previous studies with HF calculations perfectly represented the electronic structures for CeO_2, existing no localized 4*f* states. Therefore, we infer that the hybrid functionals calculation is excellent at describing the hybridization but might be unsuitable for the localized 4*f* electrons.

To avoid this problem, a widely accepted method is DFT + *U*, which introduces the Coulomb potential in a screen Hartree-Fock like manner. Considering the spin-orbital coupling (SOC) based on the second variational approach, the DFT + *U* approach opens a gap. In order to find a suitable value of U_{Pr}, we have tested the *U* with the equilibrium structure. As shown in Fig. S3a, with the U_{Pr} increasing, the occupied 4*f* states move into the low energy level while the empty 4*f* move toward the high energy level, which opens the band gap. Fig. S3b and 3c show the evolutions of the band gap and magnetic moment with the U_{Pr} change. The results derived from U_{Pr} = 6.0 eV with a band gap of 1.945 eV are very close to the previous first-principles calculations and the experimental values.

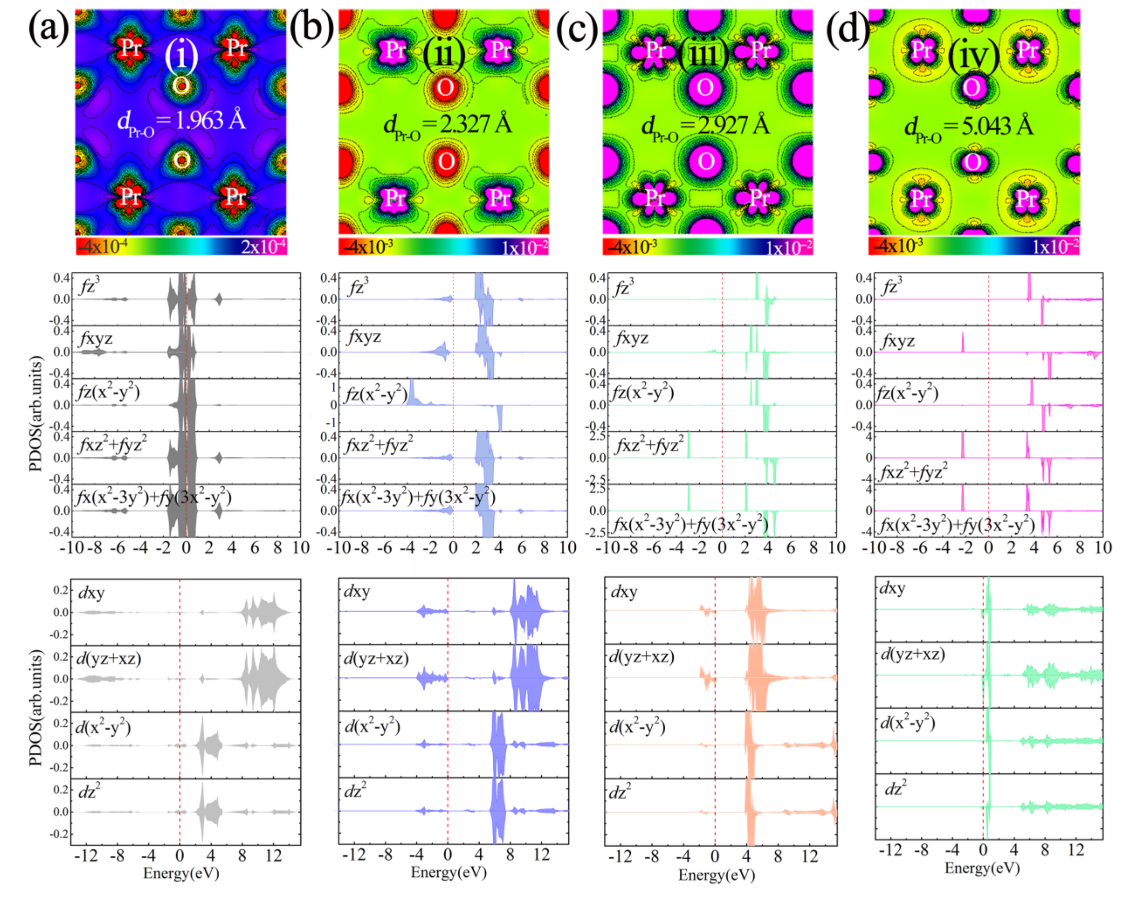

Supplementary Figure 4: The electronic properties of PrO_2 in different subareas which divided in Fig. 2(a). (**a**) - (**d**) The plots of differential spin density along with PrO_2 (111) surface and the partial DOS for 4*f* and 5*d* orbitals labeled by the value of $d_{Pr\text{-}O}$.

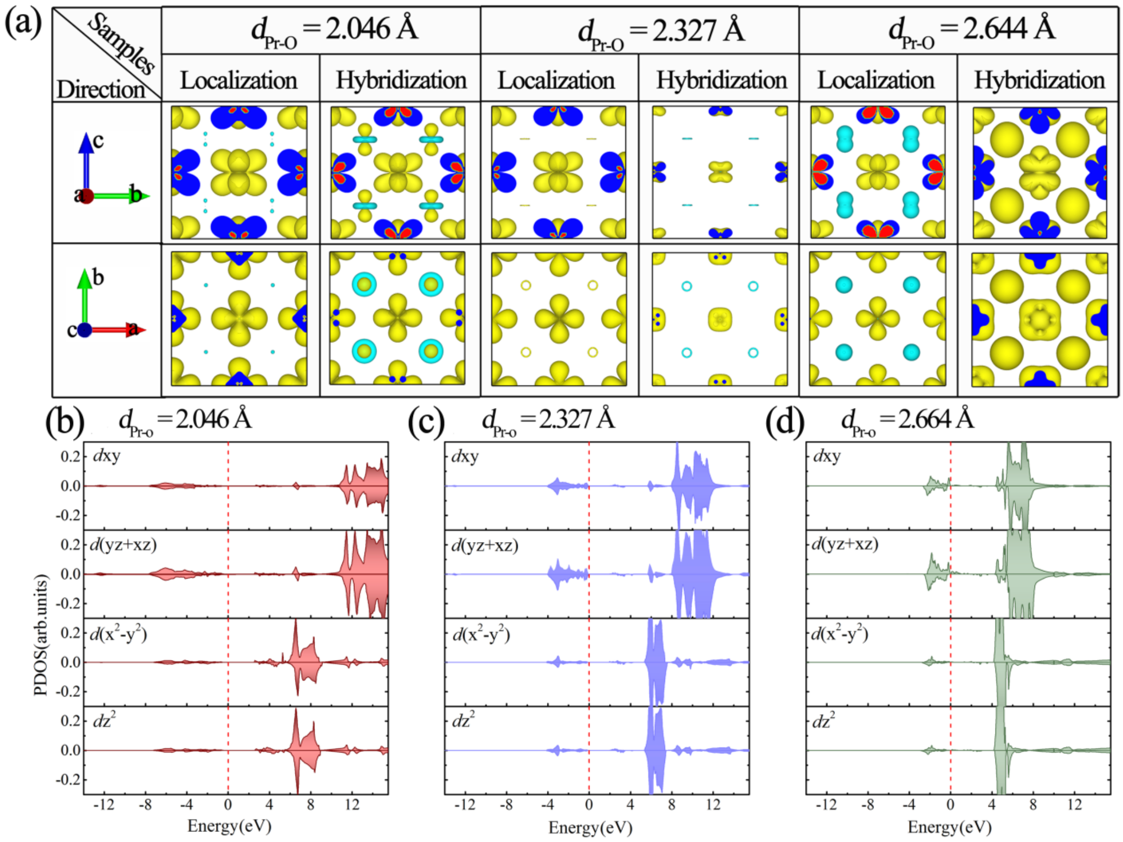

Supplementary Figure 5: The electronic properties for the representatives. (**a**) Table-like plots of the differential spin density for the change of the localized and delocalized (covalent) 4*f* electrons from two directions with the corresponding d_{Pr-O}. The value of the contour envelopes is ±0.005 $Å^{-3/2}$. (**b**) - (**c**) The PDOS of the 5*d* orbitals for PrO_2 with d_{Pr-O} = 2.046, 2.327 and 2.664 Å respectively.

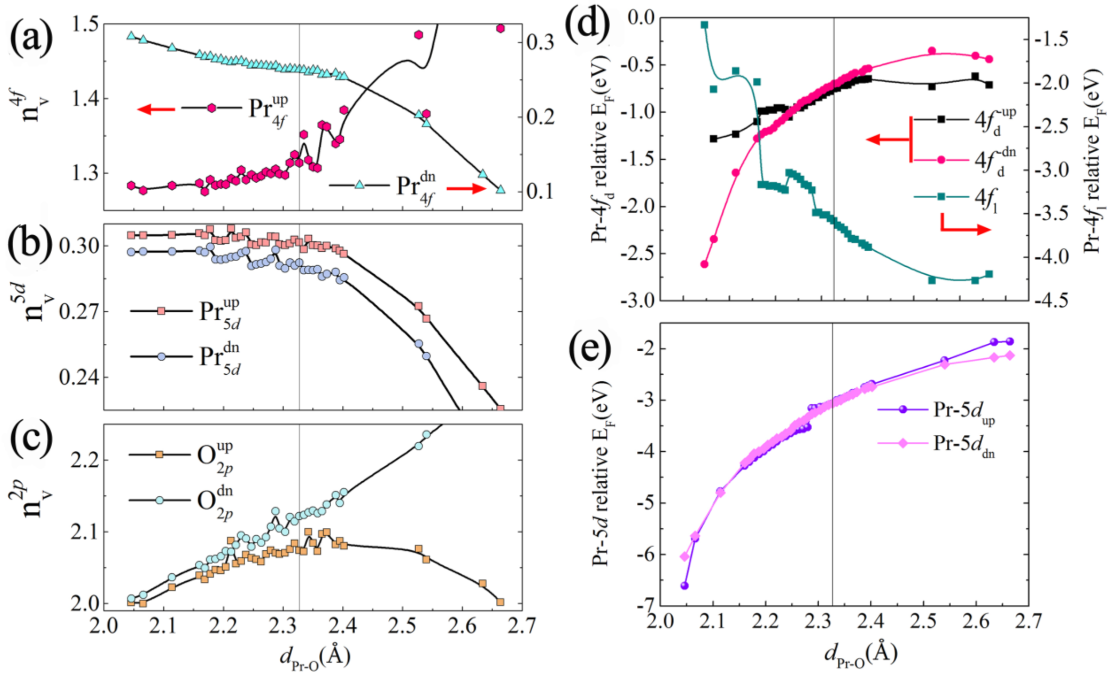

Supplementary Figure 6: The calculated orbitals' occupancies and their relative positions in area (ii). The orbital-resolved fillings of (**a**) Pr-4*f*, (**b**) Pr-5*d* and (**c**) O-2*p* valence electrons change with $d_{\text{Pr-O}}$. (**d**) and (**e**) The peak position movement of the occupied Pr-4*f* and 5*d* states, the subscript of 4f_{d} and 4f_{l} represent the delocalized and localized part, respectively.

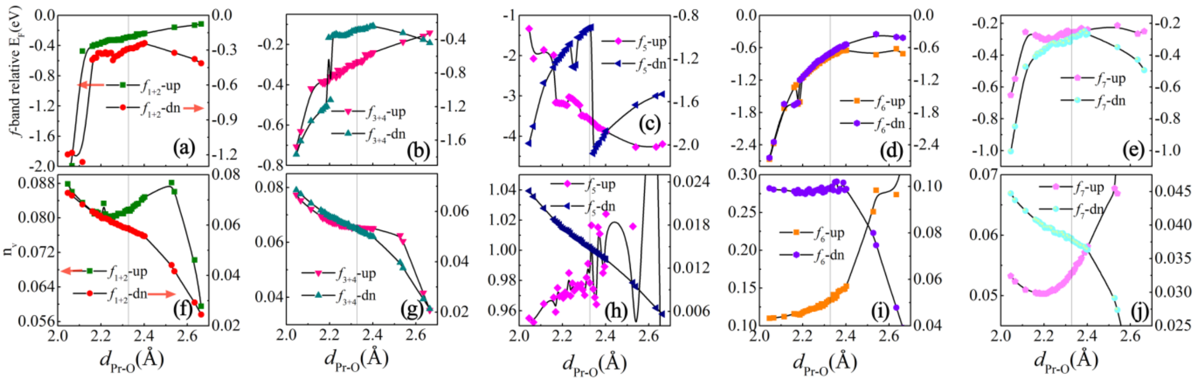

Supplementary Figure 7: The movement of the maximum peak position and the changes of orbital occupation number for the each partial 4*f* orbital in area (ii). (**a**) - (**e**) The movement of the highest peak position. (**f**) - (**j**) The evolution of orbital-resolved fillings. For

simplification, f_{1+2} represents $fx(x^2\text{-}3y^2)+fy(3x^2\text{-}y^2)$ (m = ±1), $f_{3+4} = fxz^2+fyz^2$ (m = ±3), f_5 = $fz(x^2\text{-}y^2)$ (m = +2), $f_6 = fxyz$ (m = -2) and $f_7 = fz^3$ (m = 0), respectively.

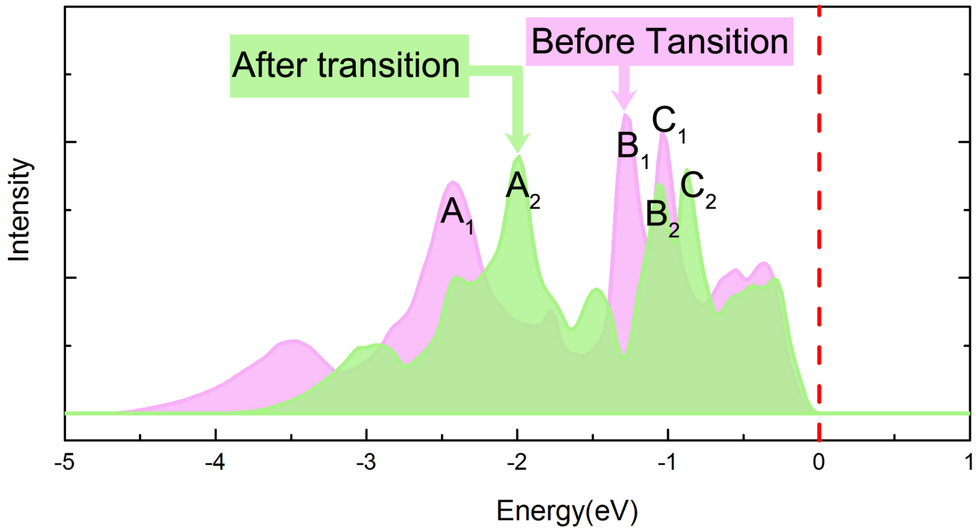

Supplementary Figure 8: The explanation of the discontinuity in Figure S7(c). There are three obvious peaks for each kind of structures, labeled by A_n, B_n and C_n.

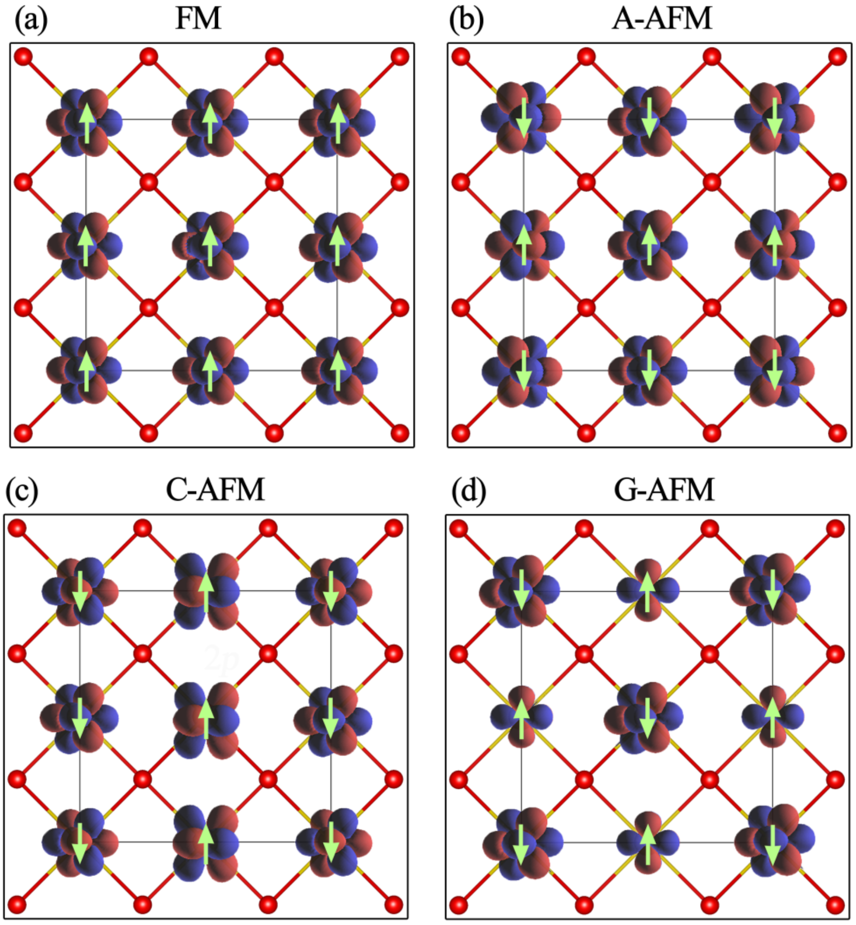

Supplementary Figure 9: The comparison of the 4*f* orbital with different magnetic orderings. (**a**) - (**d**) The localized 4*f* Wannier orbitals of the PrO_2 with different magnetic configurations, the amplitudes of the contour surface are ±0.01 Å$^{-3/2}$.

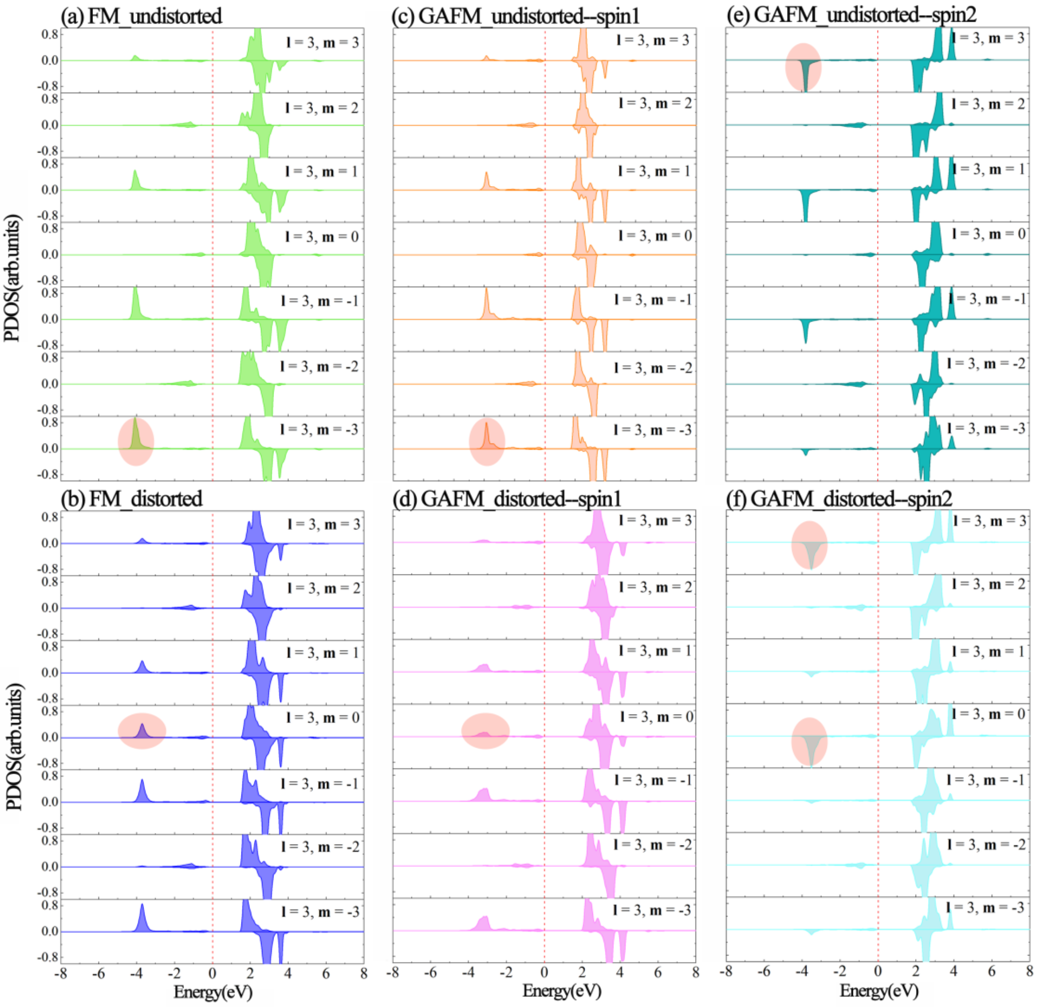

Supplementary Figure 10: The electronic comparison between the cubic and distorted PrO_2 structures. PDOS of the Pr-4*f* orbitals for each structure with different magnetic arrangements.

Supplementary Note 4: Analysis of electronic evolutions

According to the changing trend of the magnetic moment (Fig. 2a right column), we divided the dependence $d_{Pr\text{-}O}$ system for PrO_2 into four parts labeled by **i, ii**, **iii** and **iv**. In S.I. Fig.S4 we show their electronic structures for each area. Fig. S4b shows the differential spin density and partial density of states (PDOS) of 4*f* states for the ground state of PrO_2 as the representative of area **ii** (the range around the equilibrium state). In this area, the magnetic moment linearly increase and all of their configurations are tetravalent with the coexistence of intra-atomic Pr-4*f*/5*d* hybridization as covalent nature. In the area of **i**, the magnetism

disappeared is attributed to the strongly interatomic interactions which not only results from Pr-O but also from Pr-Pr and O-O. As shown in the Fig. S4a, the electronic PDOS are completely symmetric in two spin orientations. The covalency exists from the interatomic hybridization between Pr and O, but the intra-atomic 4*f*/5*d* resonance has vanished with the formation of Pr^{3+}. It could be inferred that their strong interactions in area **i** lower the ionic conductivity as the basic of electrocatalyst, while the heavy fermion behavior might be emerging there. As the lengthen $d_{\text{Pr-O}}$, the bonding character gradually transfers from covalency to ionicity. In area **iii**, the two sharp localized 4*f* orbitals as shown in Fig. S4c, which are the indication that the local magnetic moments originate from Pr-4*f* polarization and their Pr-ion also favors the trivalent configuration with trace of Pr-O hybridization. However, their large space lower the interaction between Pr and O ion, thus may facilitate the ionic conduction. In area **iv**, there is completely no interaction between each other ascribing to the large $d_{\text{Pr-O}}$, the Pr and O ions isolate in their own lattice individually, as shown in Fig. S4d. The sharp peaks of 4*f* resided below Fermi level and the small occupied 5*d* around Fermi surface demonstrate the divalent metallic character. We can infer that the activated Pr ions in area **iii** and **iv** are rather unstable due to their large attraction to the surroundings.

From Fig. S5 to Fig. S10, we supply the detailed information of electronic density for PrO_2 with different states. Fig. S5 shows the electron density of the bands in localized (-5 ~ -3 eV) and hybridized (-3 ~ 0 eV) states, and the PDOS of 5*d* electrons with $d_{\text{Pr-O}}$ = 2.046 Å, 2.327 Å and 2.664 Å. The estimations of the occupation number and the relative energy states for each O-2*p*, Pr-4*f* and 5*d* orbital are shown in Fig. S6 and S7. Fig. S8 shows the peak transition of f_5 to explain the reason of the discontinuity in Fig. S7(c). In Fig. S9, we show the localized 4*f* electron shapes with different magnetic arrangement obtained by maximally localized Wannier functions. PDOS of the split 4*f* orbitals for the prefect cubic fluorite and

oxygen distorted structure plotted in Fig. 10 shows the coupling with spin-ordering and orbital ordering in PrO_2.

Supplementary References

1. Soluyanov, A. A., Gresch, D., Wang, Z., Wu, Q., Troyer, M., Dai, X. & Bernevig, B. A. Type-II Weyl semimetals. *Nature* **527**, 495-498 (2015).
2. Zhang, H., Liu, C.-X., Qi, X.-L., Dai, X., Fang, Z. & Zhang, S.-C. Topological insulators in Bi_2Se_3, Bi_2Te_3 and Sb_2Te_3 with a single Dirac cone on the surface. *Nat. Phys*. **5**, 438-442 (2009).
3. Svane, A. & Gunnarsson, O. Transition-matal oxides in the self-interaction corrected density-functional formalism. *Phys. Rev. Lett.* **65**, 1148-1151 (1990).
4. Anisimov, V. I., Zaanen, J. & Andersen, O. K. Band theory and Mott insulators: Hubbard-*U* instead of Stonter-*I*. *Phys. Rev. B* **44**, 943-954 (1991).
5. Lichtenstein, A. I. & Katsnelson, M. I. Ab initio calculations of quasiparticle band structure in correlated systems: LDA ++ approach. *Phys. Rev. B* **57**, 6884-6895 (1998).
6. Divis, M. & Rusz, J. Calculation of crystal field for PrO_2. *J. J. Magn. Magn. Mater.* **290**, 1015-1017 (2005).
7. Da Silva, J. L. F., Ganduglia-Pirovano, M. V., Sauer, J., Bayer, V. & Kresse, G. Hybrid functionals applied to rare-earth oxides: The example of ceria. *Phys. Rev. B* **75**， 045121 (2007).
8. Kotliar, G., Savrasov, S. Y., Haule, K., Oudovenko, V. S., Parcollet, O. & Marianetti, C. A. Electronic structure calculations with dynamical mean-field theory. *Rev. Mod. Phys.* **78**, 865-951 (2006).
9. Svane, A. Dynamical mean-field theory of photoemission spectra of actinide compounds. *Solid State Comm.* **140**, 364-368 (2006).
10. Strange, P., Svane, A., Temmerman, W. M., Szotek, Z. & Winter, H. Understanding the valency of rare earths from first-principles theory. *Nature* **399**, 756-758 (1999).
11. Petit, L., Svane, A., Szotek, Z. & Temmerman, W. M. First-Principles calculations of PuO_{2-x}. *Science* **301**, 498-501 (2003).
12. Hughes, I. D., Dane, M., Ernst, A., Hergert, W., Luders, M., Poulter, J., Staunton, J. B., Svane, A., Szotek, Z. & Temmerman, W. M. Lanthanide contraction and magnetism in the heavy rare earth elements. *Nature* **446**, 650-653 (2007).
13. Tran, F., Schweifer, J., Blaha, P., Schwarz, K. & Novak, P. PBE + *U* calculations of the Jahn-Teller effect in PrO_2. *Phys. Rev. B* **77**, 085123 (2008).
14. Schwarz, K. & Blaha, P. Solid state calculations using WIEN2k. Solid state calculations using WIEN2k. *Comput. Mater. Sci.* **28**, 259-273 (2003).

15. Sjostedt, E., Nordstrom, L. & Singh, D. J. An alternative way of linearizing the augmented plane-wave method. *Solid State Comm.* **114**, 15-20 (2000).
16. Schwarz, K., Blaha, P. & Madsen, G. K. H. Electronic structure calculations of solids using the WIEN2k package for material sciences. *Comput. Phys. Commun* **147**, 71-76 (2002).
17. Murnaghan F. D. The Compressibility of Media under Extreme Pressures. *Proc. Natl. Acad. Sci. USA* **30**, 244-247 (1944).
18. Perdew, J. P. & Yue, W. Accurate and simple density functional for the electronic exchange energy: generalized gradient approximation. *Phys. Rev. B* **33**, 8800-8802 (1986).
19. Perdew, J. P., Burke, K. & Ernzerhof, M. Generalized gradient approximation made simple. *Phys. Rev. Lett.* **77**, 3865-3868 (1996).
20. Perdew, J. P., Ruzsinszky, A., Csonka, G. I., Vydrov, O. A., Scuseria, G. E., Constantin, L. A., Zhou, X. L. & Burke, K. Restoring the density-gradient expansion for exchange in solids and surfaces. *Phys. Rev. Lett.* **100**, 136406 (2008).
21. Kresse, G. & Furthmuller, J. Efficient iterative schemes for ab initio total-energy calculations using a plane-wave basis set. *Phys. Rev. B* **54**, 11169-11186 (1996).
22. Kresse, G. & Hafner, J. Ab-initio molecular-dynamics for open-shell transition-matals. *Phys. Rev. B* **48**, 13115-13118 (1993).
23. Ėvarestov, R. A. *Quantum chemistry of solids : the LCAO first principles treatment of crystals* (Springer, Berlin, 2007).
24. Gerward, L., Olsen, J. S., Petit, L., Vaitheeswaran, G., Kanchana, V. & Svane, A. Bulk modulus of CeO_2 and PrO_2: An experimental and theoretical study. J. Alloys Compd. 400, 56-61 (2005).
25. Tran, F. & Blaha, P. Implementation of screened hybrid functionals based on the Yukawa potential within the LAPW basis set. Phys. Rev. B 83, 235118 (2011).
26. Mostofi, A. A., Yates, J. R, Lee, Y. –S., Souza, I., Vanderbilt, D. & Marzari, N. Wannier90: A tool for obtaining maximally-localised Wannier functions. *Comput. Phys. Comm.* **178**, 685-699 (2008), http://www.wannier.org.
27. Wannier, G. H. The structure of electronic excitation levels in insulating crystals. *Phys. Rev.* **52**, 0191-0197 (1937).
28. Kuneš, J., Arita, R., Wissgott, P., Toschi, A., Ikeda, H. & Held, K. Wien2wannier: From linearized augmented plane waves to maximally localized Wannier functions. *Comput. Phys. Comm.* **181**, 1888-1895 (2010).
29. van der Kolk, E. & Dorenbos, P. Systematic and material independent variation of electrical, optical, and chemical properties of Ln materials over the Ln series (Ln = La, Ce, Pr,..., Lu). *Chem. Mater.* **18**, 3458-3462 (2006).
